# Major depletion of SOX2^+^ stem cells in the adult pituitary is not restored which does not affect hormonal cell homeostasis and remodelling

**DOI:** 10.1038/s41598-017-16796-2

**Published:** 2017-12-05

**Authors:** Heleen Roose, Benoit Cox, Matteo Boretto, Conny Gysemans, Annelies Vennekens, Hugo Vankelecom

**Affiliations:** 1 Department of Development and Regeneration, Cluster of Stem Cell and Developmental Biology (SCDB), Unit of Stem Cell Research, KU Leuven (University of Leuven), Leuven, Belgium; 2 Department of Chronic Diseases, Metabolism and Ageing, Clinical and Experimental Endocrinology, KU Leuven (University of Leuven), Leuven, Belgium

## Abstract

The pituitary gland contains SOX2-expressing stem cells. However, their functional significance remains largely unmapped. We investigated their importance by depleting SOX2^+^ cells through diphtheria toxin (DT)-mediated ablation. DT treatment of adult Sox2^CreERT2/+^;R26^iDTR/+^ mice (after tamoxifen-induced expression of DT receptor in SOX2^+^ cells) resulted in 80% obliteration of SOX2^+^ cells in the endocrine pituitary, coinciding with reduced pituisphere-forming activity. Counterintuitively for a stem cell population, the SOX2^+^ cell compartment did not repopulate. Considering the more active phenotype of the stem cells during early-postnatal pituitary maturation, SOX2^+^ cell ablation was also performed in 4- and 1-week-old animals. Ablation grade diminished with decreasing age and was accompanied by a proliferative reaction of the SOX2^+^ cells, suggesting a rescue attempt. Despite this activation, SOX2^+^ cells did also not recover. Finally, the major SOX2^+^ cell depletion in adult mice did not affect the homeostatic maintenance of pituitary hormonal cell populations, nor the corticotrope remodelling response to adrenalectomy challenge. Taken together, our study shows that pituitary SOX2^+^ fail to regenerate after major depletion which does not affect adult endocrine cell homeostasis and remodelling. Thus, pituitary SOX2^+^ cells may constitute a copious stem cell reserve or may have other critical role(s) still to be clearly defined.

## Introduction

The pituitary gland plays a pivotal role in the endocrine system and governs essential physiological processes like growth, metabolism, puberty, reproduction and stress response. The gland consists of different lobes, the anterior pituitary (AP), intermediate lobe (IL) and posterior pituitary. The AP represents the major endocrine part of the gland producing the key hormones prolactin (PRL), growth hormone (GH), adrenocorticotropic hormone (ACTH), thyroid-stimulating hormone (TSH), luteinizing hormone (LH) and follicle-stimulating hormone (FSH). Because of its central role, malfunctioning of the pituitary has critical consequences for body physiology, causing, amongst others, diabetes, cardiovascular disease, osteoporosis, infertility and/or psychological disorders^1^. Pituitary hormonal cell populations must therefore be maintained in a controlled and balanced manner.

Postnatal turnover of tissues classically includes the generation of new mature cells from resident stem cells. In the pituitary, stem cells have been identified, displaying as central characteristic the expression of the stemness regulator SRY-related HMG box transcription factor 2 (SOX2)^2–5^. Despite their identification about 10 years ago, the functional role of the stem cells in the postnatal gland is far from clear. Following pituitary damage as inflicted by transgenic endocrine cell ablation, the SOX2^+^ stem cell compartment becomes activated; acute expansion of the SOX2^+^ cell population and co-expression of the ablated hormone supports their involvement in the regenerative response that is unfolding upon injury^6–8^. Recent genetic lineage tracing studies revealed that SOX2^+^ cells contribute to the different hormonal cell types during postnatal homeostatic turnover but only at low frequency, while displaying long-term persistence suggesting a long-lived character and (slow) self-renewal activity^9,10^. In addition, pituitary SOX2^+^ cells have been suggested to act as signalling centres, particularly in disease conditions like tumorigenesis in which paracrine signalling from (activated) SOX2^+^ cells have the capacity to promote tumour development in the gland^9,11^. Here, we aimed at investigating the functional significance of SOX2^+^ cells in the postnatal pituitary by ablating these cells using a transgenic diphtheria toxin (DT)-mediated system. In addition, we explored the self-regenerating capacity of the SOX2^+^ pituitary stem cells. Our study shows that SOX2^+^ cells of the adult pituitary do not restore their own cell compartment after major depletion, which does not affect the maintenance of the different hormonal cell populations during homeostasis, nor the endocrine cell remodelling as triggered by adrenalectomy.

## Results

### SOX2^+^ cells do not repopulate after major ablation in the adult pituitary

**Figure 1:**
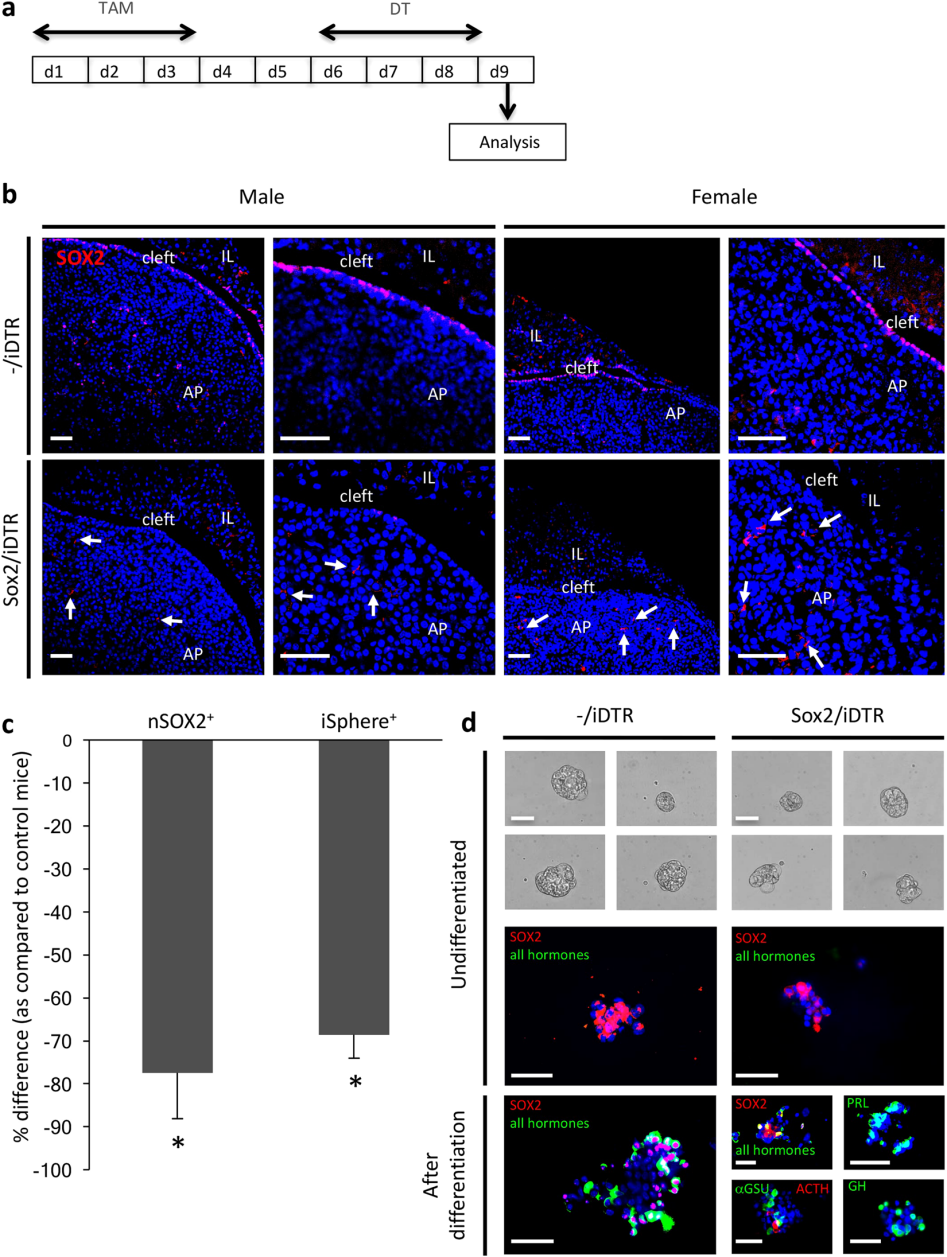
SOX2^+^ cell ablation in the pituitary of adult mice. (**a)** Time schedule of TAM/DT injections and pituitary analysis. (**b)** Pituitary vibratome sections isolated from adult, male and female control (-/iDTR) and Sox2/iDTR mice injected with TAM/DT and analysed for SOX2 (red) immediately after treatment (day 9, d9). Representative pictures are shown, the nucleus being labelled with TOPRO3 (blue). Scale bar: 50 μm. AP, anterior pituitary; IL, intermediate lobe. Surviving SOX2^+^ cells with immunoreactive signal in the cytoplasm (cSOX2^+^ cells) are indicated (arrows). **(c)** Percent decrease in nSOX2^+^ cells (SOX2^+^ signal in the nucleus) and in sphere-initiating (iSphere^+^) cells in the AP at d9 after TAM/DT injection of adult Sox2/iDTR mice as compared to -/iDTR control mice. Bars represent mean ± SEM (n = 4). *p < 0.05 (versus control). (**d)** Primary spheres at day 6 after seeding AP cells from adult Sox2/iDTR and -/iDTR control mice immediately after DT injection (d9). Primary (undifferentiated) spheres (day 6) were subjected to differentiation on Matrigel (for another 7 days) and immunostained before and after differentiation for SOX2 (red) and hormones (as indicated; green). Representative pictures are shown. Scale bar:  50 μm.

To investigate the role of the SOX2^+^ cells in the adult pituitary, we embarked on their ablation by using the DT/inducible DT receptor (iDTR) system. The iDTR mouse was crossed to the SOX2CreERT2 mouse in which CreERT2 is expressed under control of the endogenous *Sox2* promoter and activated by tamoxifen (TAM). Mice were treated with TAM and DT according to an optimized schedule (see Methods and Fig. 1a).

The pituitary of TAM/DT-injected adult (8- to 12-week-old) Sox2^CreERT2/+^;R26^iDTR/+^ mice (further referred to as ‘Sox2/iDTR’), as isolated immediately after DT treatment (i.e. at d9; see Fig. 1a), did morphologically not differ from the glands of control Sox2^+/+^;R26^iDTR/+^ littermates (further referred to as ‘-/iDTR’) (Supplementary Fig. S1). However, immunofluorescence examination of pituitary sections showed a clear loss in SOX2^+^ cells when compared to controls, both in male and female mice (Fig. 1b; Supplementary Fig. S2). The DT treatment induced cell apoptosis in the SOX2^+^ cells as shown by cleaved caspase 3 (CC3) immunostaining (Supplementary Fig. S2). Especially the SOX2^+^ cells in the marginal-zone (MZ) stem cell niche (i.e. around the cleft which is the remnant lumen of the embryonic pituitary) were found to be eliminated, coinciding with a major decrease in MZ cells expressing E-cadherin and SOX9, two other pituitary stem cell markers^2,9,10,12^ (Supplementary Fig. S2). In accordance, gene expression levels in the AP of these markers (and another pituitary stem cell marker as found in rat, *Cxadr* or coxsackie virus and adenovirus receptor^13^) were reduced (Supplementary Fig. S2). Within the AP parenchyma, SOX2^+^ cells were still observed. The remaining parenchymal SOX2^+^ cells often represented cells with SOX2-immunoreactive signal in the cytoplasm, not in the nucleus (Fig. 1b; Supplementary Fig. S2). Quantification using AP cell cytospin samples (pooled from male and female mice, see Methods) indeed confirmed that the cells with SOX2^+^ signal in the nucleus (further abbreviated as nSOX2^+^ cells) showed a major drop of ∼80% in the Sox2/iDTR mice (Fig. 1c), whereas the cells with SOX2^+^ signal in the cytoplasm (cSOX2^+^ cells) were not significantly affected (Supplementary Fig. S2). Absence of further active transcription of the *Sox2* gene in these cSOX2^+^ cells may explain their escape from *Sox2* promoter-driven ablation. In line with this hypothesis, CRE expression (as driven by the endogenous *Sox2* promoter and translocated to the nucleus by TAM) was predominantly found in the cells with SOX2^+^ signal in the nucleus (Supplementary Fig. S2). In agreement with the drop in the nSOX2^+^ stem cells, stem cell-linked sphere-inducing capacity within the AP cell population was also strongly reduced (Fig. 1c). Multipotent differentiation capacity of the remaining sphere-inducing cells toward hormonal cells was still functional as tested for distinct hormonal cell lineages (i.e. ACTH^+^, GH^+^, PRL^+^ and αGSU^+^ cells, the latter encompassing the TSH^+^ thyrotropes and the LH^+^/FSH^+^ gonadotropes; Fig. 1d). Of note, the *Sox2* hemizygous genotype as occurring in the Sox2/iDTR mouse (i.e. knock-in of the *CreERT2* transgene in one *Sox2* allele resulting in *Sox2* haploinsufficiency) did not in itself affect the number of SOX2^+^ cells in the AP (Supplementary Fig. S3).

**Figure 2:**
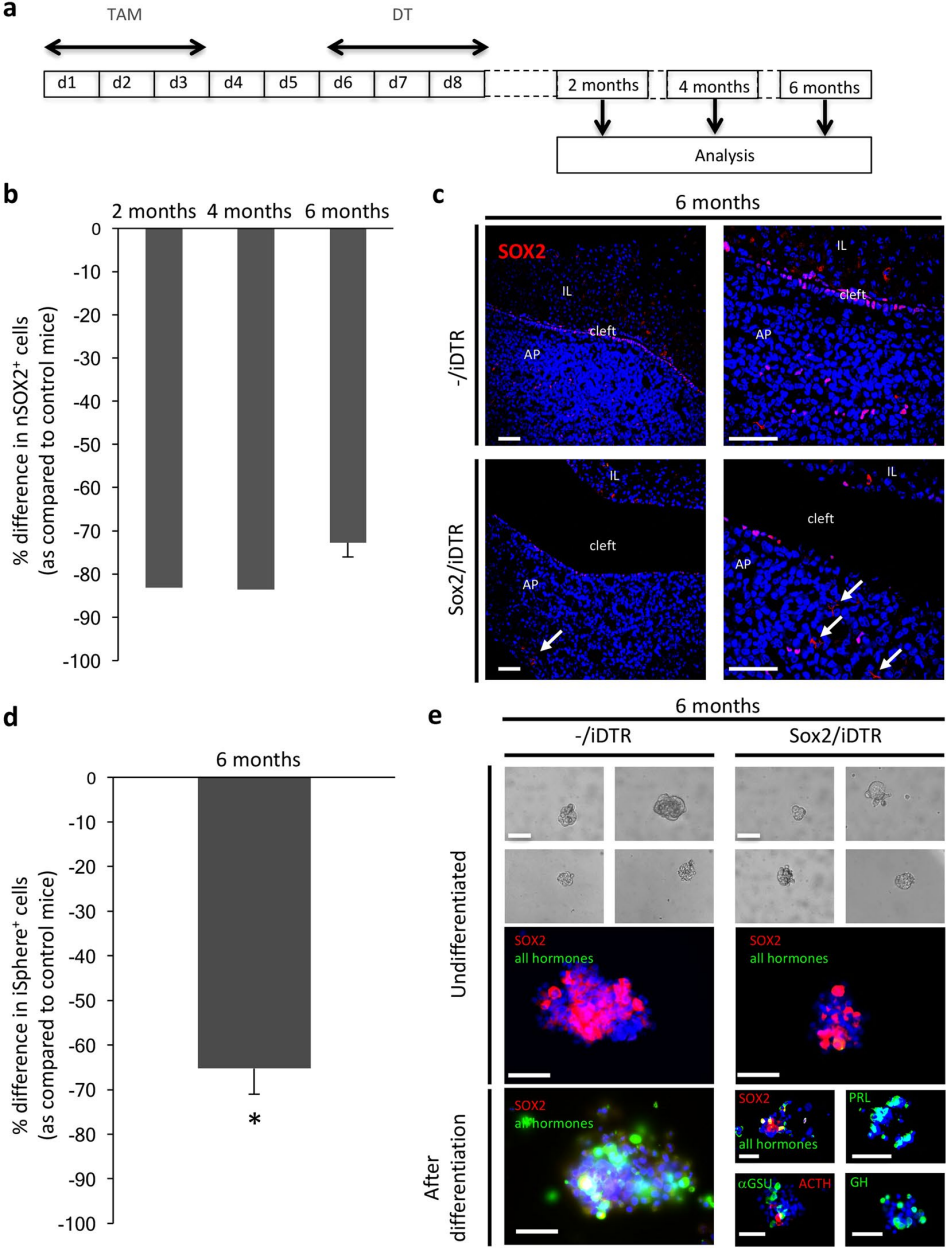
SOX2^+^ cells do not repopulate after major ablation in the adult pituitary. (**a)** Time schedule of TAM/DT injections and pituitary analysis. (**b)** Percent decrease in nSOX2^+^ cells in the AP after TAM/DT injection of adult Sox2/iDTR mice as compared to -/iDTR control mice at the different time points indicated. Bars represent mean (± SEM for n ≥ 3) (2 months: n = 1; 4 months: n = 2; 6 months: n = 4). (**c)** Pituitary vibratome sections isolated from adult control (-/iDTR) and Sox2/iDTR mice injected with TAM/DT and analysed for SOX2 (red) 6 months after treatment. Representative pictures are shown, the nucleus being labelled with TOPRO3 (blue). Scale bar: 50 μm. cSOX2^+^ cells are indicated (arrows). (**d)** Percent decrease in iSphere^+^ cells 6 months after TAM/DT injection of adult Sox2/iDTR mice as compared to -/iDTR control mice. Bar represents mean ± SEM (n = 4). *p < 0.05 (versus control). (**e**) Primary spheres at day 6 after seeding AP cells from adult Sox2/iDTR and -/iDTR control mice 6 months after DT injection. Primary (undifferentiated) spheres were subjected to differentiation on Matrigel and immunostained before and after differentiation for SOX2 (red) and hormones (as indicated; green). Representative pictures are shown. Scale bar: 50 μm.

Next, we examined whether the SOX2^+^ cell compartment repopulated after its major depletion and therefore determined SOX2^+^ cell proportions 2, 4 and 6 months after ending the DT treatment (Fig. 2a). Intriguingly, the nSOX2^+^ stem cell population did not restore (Fig. 2b,c; ablation after 6 months *versus* d9: non-significant, see Supplementary Table S1), while the cSOX2^+^ cell proportion did not significantly change at all time points analysed when compared to control -/iDTR AP (Supplementary Fig. S2). Sphere-forming capacity also remained largely diminished after 6 months, while multipotent differentiation capacity of the lasting sphere-initiating cells was again not affected (Fig. 2d,e). Of note, the mice retained a healthy appearance during the whole 6-month period, in contrast to an earlier study in which lethality was observed after 2 weeks of continuously destroying the (dividing) SOX2^+^ cells with the thymidine kinase (TK)/ganciclovir (GCV) system, reportedly caused by disrupted epithelial integrity and ulcer development in the stomach^4^. However, when GCV treatment was stopped once animals became morbid (i.e. after 1 week), gradual recovery was observed, coinciding with a repopulation of the SOX2^+^ cells in the stomach^4^. In our study, the TAM/DT treatment of Sox2/iDTR mice also caused an acute SOX2^+^ cell ablation in the stomach (as analysed at d9; Supplementary Fig. S4), but SOX2^+^ cell recovery was observed in this highly renewing tissue (as shown for the 6-month time point; Supplementary Fig. S4), most likely explaining the non-toxic impact on the animals.

Taken together, SOX2^+^ stem cells of the adult pituitary do not regenerate after major ablation, which is intriguing and counterintuitive for a stem cell population and which suggests that the adult pituitary stem cells do not need to restore to full number size for adequate basal pituitary functioning.

### SOX2^+^ cells of the early-postnatal pituitary react to their ablation but also do not repopulate

During the neonatal and early-postnatal period, the mouse pituitary undergoes active growth and maturation with significant expansion of the hormonal cell populations^14–16^. We previously observed that the pituitary stem cell compartment is enlarged in the first weeks after birth and shows functional and molecular signs of activation^16^. In agreement, recent SOX2^+^ lineage tracing studies revealed a higher number of descendant hormonal cells when tracing was initiated shortly after birth as compared to later in life^9,10^. Therefore, we examined whether the more active pituitary SOX2^+^ stem cells as present in younger animals could recover after DT-mediated ablation.

**Figure 3:**
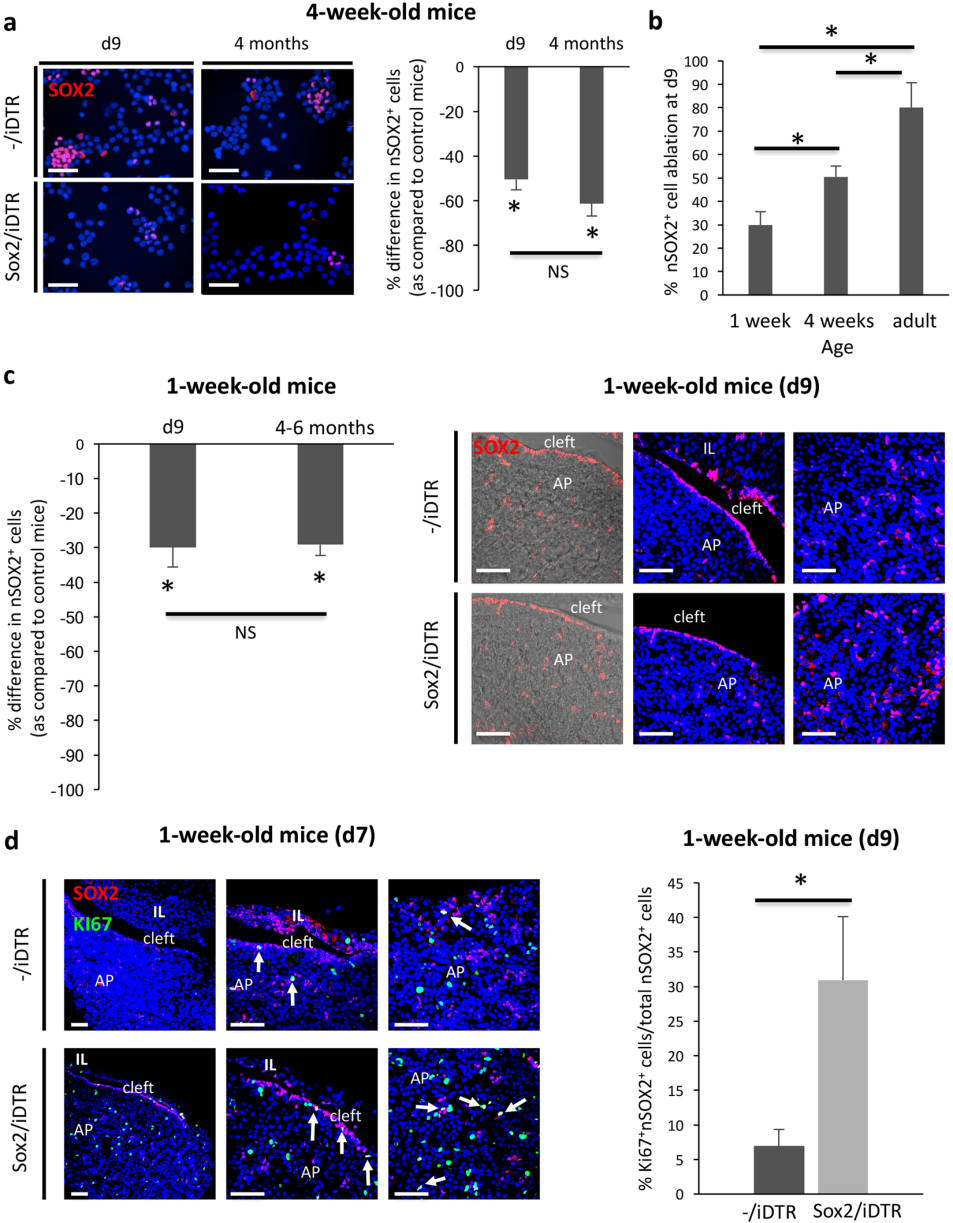
SOX2^+^ cell ablation in the pituitary of early-postnatal mice: proliferative reaction but no regeneration. (**a)** *Left*: Cytospin samples of AP cells immunostained for SOX2 (red) from 4-week-old -/iDTR control and Sox2/iDTR mice immediately after DT treatment (d9) and 4 months later. Nuclei are labelled with DAPI (blue). Representative pictures are shown. Scale bar:  50 μm. *Right*: Bar graph showing the percent decrease (mean ± SEM; d9: n = 4; 4 months: n = 3) in nSOX2^+^ cells in the AP after TAM/DT injection of 4-week-old Sox2/iDTR mice as compared to -/iDTR control mice at the different time points indicated. *p < 0.05 (versus control); NS, non-significant. (**b)** Percent ablation of nSOX2^+^ cells in the AP immediately (d9) after TAM/DT injection of Sox2/iDTR mice at different ages, as compared to -/iDTR control mice. Bars represent mean ± SEM (1-week old: n = 3; 4-week-old: n = 4; adult or 8–12 weeks old: n = 4). *p < 0.05. (**c)** *Left*: Percent decrease in nSOX2^+^ cells in the AP after TAM/DT injection of 1-week-old Sox2/iDTR mice as compared to -/iDTR control mice at the time points indicated. Bars represent mean ± SEM (n = 3). *p < 0.05 (versus control). NS, non-significant. *Right*: Pituitary vibratome sections isolated from -/iDTR and Sox2/iDTR mice injected at 1 week of age with TAM/DT and analysed for SOX2 (red) immediately after treatment (d9). Representative combined immunofluorescent/brightfield-contrast and immunfluorescent pictures are shown, the nucleus being labelled with TOPRO3 (blue). Scale bar: 50 μm. (**d)** *Left*: Pituitary vibratome sections isolated from -/iDTR and Sox2/iDTR mice injected at 1 week of age with TAM/DT and analysed for SOX2 (red) and Ki67 (green) at d7 (i.e. after the first day of DT injection, see Fig. 1a). Representative pictures are shown, the nucleus being labelled with TOPRO3 (blue). Scale bar: 50 μm. Double nSOX2^+^/Ki67^+^ cells are indicated (arrows). *Right*: Bar graph showing the proportion of Ki67^+^/nSOX2^+^ within the nSOX2^+^ cell population (mean ± SEM; n = 3) in TAM/DT-injected 1-week old Sox2/iDTR mice (light grey) as compared to -/iDTR control mice (dark grey), analysed immediately after TAM/DT injection (d9). *p < 0.05.

First, we investigated 4-week-old mice and applied the TAM/DT injection schedule as described above. Analysis at d9 revealed a clear drop in pituitary nSOX2^+^ cells in the Sox2/iDTR mice (pooled male and female), however smaller than in adult mice (Fig. 3a,b). Again, cSOX2^+^ cells did not significantly change (Supplementary Fig. S5). Despite this smaller ablation grade as compared to adult mice, the number of nSOX2^+^ cells did also not restore as analysed 4 months later (Fig. 3a).

Then, we selected the earliest feasible time point of TAM/DT injection (meaning, without lethality), and treated 1-week-old pups (pooled male and female). Pituitary morphology did not visibly change (Supplementary Fig. S6). Ablation of nSOX2^+^ cells was observed, but again lower than in adults and also lower than in 4-week-old mice (Fig. 3b,c), while cSOX2^+^ cells did not significantly alter (Supplementary Fig. S6). DT-induced apoptosis was detected in the pituitary but not at high extent (as analysed at d7 and d9; Supplementary Fig. S6), which may suggest that the TAM/DT system shows low(-er) efficacy at this neonatal age, for instance because of less efficient delivery of the compounds in the very young animals and/or low expression of CRE (as indeed observed, see Supplementary Fig. S6). In addition, the more active SOX2^+^ stem cells of the neonatal pituitary may attempt to counteract their elimination. In line with this possibility, a clear increase in proliferating (Ki67^+^) nSOX2^+^ cells was observed in the Sox2/iDTR pituitary immediately after ablation when compared to control -/iDTR mice (Fig. 3d and Supplementary Fig. S6). Cycling SOX2^+^ cells particularly emerged in the MZ, which were practically not observed in the control pituitary. Moreover, Ki67 was only detected in the nSOX2^+^ cells and not in cSOX2^+^ cells (data not shown). A proliferative reaction of the nSOX2^+^ cells was also observed in the pituitary of 4-week-old mice (Supplementary Fig. S6), whereas proliferation within the SOX2^+^ cell compartment of adult pituitary did not change immediately after ablation, remaining almost undetectable as similar to control mice (data not shown). Nevertheless, despite the proliferative response of the nSOX2^+^ cells in the neonatal mice, their number was not restored when analysed 4–6 months later (Fig. 3c) at which time point virtually no proliferating nSOX2^+^ cells were observed anymore (Supplementary Fig. S6), in line with the static phenotype of the gland at adult age. cSOX2^+^ cells were again not different at 4–6 months (Supplementary Fig. S6).

Taken together, our data indicate that the SOX2^+^ stem cells in the young pituitary actively react to the destruction process within their compartment by acutely proliferating, plausibly to immediately restore their cell population and/or to rescue the pituitary maturation process. Despite this reaction, the affected SOX2^+^ cell population did not repopulate, again suggesting that the stem cells do not need to restore to full number size for adequate basal pituitary functioning.

### Proportions of hormonal cell populations do not change after SOX2^+^ cell ablation in the postnatal pituitary

**Figure 4:**
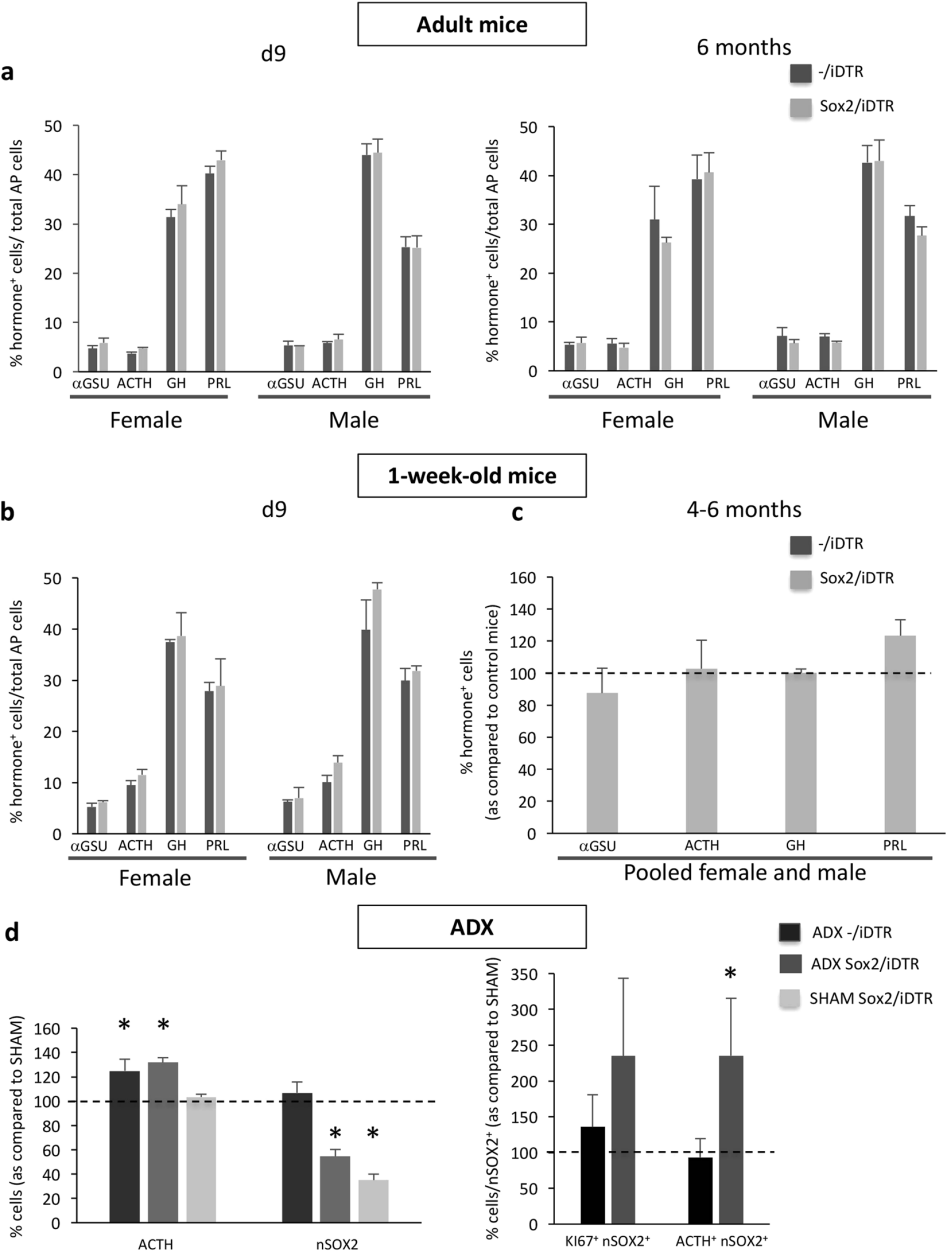
SOX2^+^ cell ablation does not affect pituitary hormonal cell populations. (**a**) Proportion of hormone^+^ cells as quantified in immunostained AP cell cytospin samples obtained from adult TAM/DT-injected female and male control -/iDTR mice (dark grey) and Sox2/iDTR mice (light grey), analysed 1 day (i.e. at d9; *left*) and 6 months (*right*) later. Bars represent mean ± SEM (females: n = 4 and 3, respectively; males: n = 3). No significant differences were observed (see Supplementary Table S1). (**b)** Proportion of hormone^+^ cells as quantified in immunostained AP cell cytospin samples obtained from 1-week-old TAM/DT-injected female and male control -/iDTR mice (dark grey) and Sox2/iDTR mice (light grey), analysed 1 day later (d9). Bars represent mean ± SEM (females: n = 3; males: n = 3). No significant differences were observed (see Supplementary Table S1). (**c)** Proportion of hormone^+^ cells as quantified in immunostained AP cell cytospin samples obtained from 1-week-old TAM/DT-injected Sox2/iDTR mice (as compared to control -/iDTR mice, set as 100%), analysed 4–6 months later. Bars represent mean ± SEM (from pooled female and male mice: n = 3). No significant differences were observed (see Supplementary Table S1). (**d)** *Left*: Proportion of cells immunoreactive for ACTH and nSOX2 as indicated, quantified in immunostained AP cell cytospin samples obtained from adult TAM/DT-injected -/iDTR mice after ADX (black bars), adult TAM/DT-injected Sox2Cre/iDTR mice after ADX (dark grey bars) and adult TAM/DT-injected Sox2Cre/iDTR mice after SHAM operation (light grey bars) (as compared to control -/iDTR mice after SHAM operation, set as 100%). Bars represent mean ± SEM (from pooled female and male mice, n = 3–5). *p < 0.05 (see Supplementary Table S1). *Right*: Proportion of cells double-immunoreactive for the factors as indicated, quantified in immunostained AP cell cytospin samples obtained from adult TAM/DT-injected -/iDTR mice after ADX (black bars) and adult TAM/DT-injected Sox2Cre/iDTR mice after ADX (dark grey bars) (as compared to control -/iDTR mice after SHAM operation, set as 100%). Double immunostainings were not performed for adult TAM/DT-injected Sox2Cre/iDTR mice after SHAM operation. Bars represent mean ± SEM (from pooled female and male mice, n = 3–5). *p < 0.05 (see Supplementary Table S1).

Turnover in adult tissues may involve several mechanisms, including the generation of new cells by the resident stem cells. Therefore, we addressed the question whether the major depletion of SOX2^+^ cells in the adult pituitary had an impact on the endocrine cell composition of the gland, either immediately after TAM/DT treatment (d9) or 4–6 months later. At both time points, the proportions of the different hormone^+^ cell populations were not changed when compared to control -/iDTR pituitaries, independent of gender (Fig. 4a). Thus, absence of a major part of SOX2^+^ cells in the adult pituitary does not affect AP endocrine cell homeostasis. Other mechanisms may compensate to maintain the hormonal cell populations in the absence of SOX2^+^ cell-derived endocrine cell generation. First, other stem cell (sub-)populations, if present, may make up for the loss of the SOX2^+^ stem cell compartment. We analysed other pituitary stem cell markers and found that SOX9^+^ and E-cadherin^+^ cells were also not restored at 6 months after ablation, and that the gene expression levels remained reduced (Supplementary Fig. S2). The formation of new endocrine cells may also be compensated for by higher proliferation and transdifferentiation of mature hormonal cells. We investigated these possibilities within the two largest hormonal cell populations, i.e. the GH^+^ and PRL^+^ lineages. Only few cells were double GH^+^/Ki67^+^ (Supplementary Fig. S7) and PRL^+^/Ki67^+^ (data not shown) in the control -/iDTR pituitary which did not alter after SOX2^+^ cell ablation. Moreover, double GH^+^/PRL^+^ cells, which would represent intermediate stages in transdifferentiation events between both cell types, did not change in Sox2/iDTR pituitary as compared to control (Supplementary Fig. S7). On the other hand, enhanced differentiation of the remaining SOX2^+^ cells may play a role in the maintenance of the hormonal cell populations. However, the percentage of SOX2^+^ cells that co-expressed GH (Supplementary Fig. S7) and PRL (data not shown) did not change in adult mice.

The lower ∼30% SOX2^+^ cell depletion at neonatal age did also not have any impact on the hormonal cell populations (Fig. 4b,c), despite the pronounced growth and differentiation process known to occur in the gland during the early-postnatal period. However, the proportion of GH^+^/SOX2^+^ and PRL^+^/SOX2^+^ cells within the total AP and within the large group (∼70%) of remaining SOX2^+^ cells increased after SOX2^+^ cell ablation (Supplementary Fig. S7). This observation, together with the enhanced proliferative activity within the remaining SOX2^+^ cell compartment in the neonatal pituitary (see above), supports the occurrence of an activation response of the young SOX2^+^ stem cells, possibly to sustain the early-postnatal differentiation wave.

### Major SOX2^+^ cell depletion in the adult pituitary does not affect corticotrope expansion upon adrenalectomy challenge

The large depletion of SOX2^+^ stem cells in the adult pituitary did not affect hormonal cell proportions in steady-state conditions. To investigate whether SOX2^+^ cell ablation has an impact in more active endocrine cell remodelling conditions of the pituitary, adrenalectomy (ADX) was performed. ADX is known to cause a prompt increase (∼20%) in the number of ACTH^+^ corticotropes in the pituitary, due to the disappearance of negative feedback from the target organ^10,17^. Previous studies provided circumstantial evidence that pituitary stem cells may be involved through a mitotic wave followed by differentiation into the new hormonal cells^17^. A recent study provided more direct evidence using SOX9^+^ lineage tracing, showing that part (∼20%) of the newly formed corticotropes are derived from pituitary (SOX9^+^) stem cells^10^. Here, we investigated whether the major SOX2^+^ cell depletion as obtained in adult mice had an effect on the rise of ACTH^+^ cells after ADX. We found that ACTH^+^ cells increased in number by ∼20% after ADX (as compared to SHAM operation), independent of prior SOX2^+^ cell ablation (Fig. 4d). Interestingly, the nSOX2^+^ cells remaining after DT-mediated depletion significantly increased (∼1.5-fold) in number after ADX, indicating that these residual nSOX2^+^ cells react to the ADX-imposed endocrine challenge in the pituitary. In accordance, the proliferating fraction within the remaining nSOX2^+^ cell population showed an increase by ∼2.5-fold (although not statistically significant; Fig. 4d and Supplementary Table S1). Moreover, whereas ADX did not augment the proportion of double nSOX2^+^/ACTH^+^ cells within the whole nSOX2^+^ cell population as present in ‘normal’ mice (in comparison with SHAM-operated mice), this proportion significantly (2.4-fold) increased within the nSOX2^+^ cell compartment remaining after DT-mediated depletion (Fig. 4d).

Taken together, the large depletion of SOX2^+^ stem cells in the adult pituitary does not affect hormonal cell remodelling upon endocrine challenge, at least as examined for corticotrope expansion after ADX.

## Discussion

Stem cells in general play a key role in postnatal homeostatic turnover of their tissue and in repair of injury by cell regeneration. However, knowledge on the role of the stem cells in the pituitary gland, as marked by SOX2 expression, is at present very limited. The pituitary SOX2^+^ cells appear involved in regeneration after hormonal cell-ablation injury^6–8^, and generate cells of the different hormonal cell lineages during postnatal homeostatic turnover, although at low number^9,10^. We here applied DT-mediated SOX2^+^ cell ablation to investigate the stem cells’ regenerative response and the influence on endocrine cell populations. A previous study examined the impact of overall SOX2^+^ cell ablation in several tissues, but effects in the pituitary were not described^4^. In this particular study, ablation was achieved using the TK/GCV system, signifying that only dividing SOX2^+^ cells were obliterated. Given the low turnover rate of the adult pituitary and the mainly non-proliferative nature of its SOX2^+^ cells^2,6,9,10,12^, it is highly likely that SOX2^+^ cells of the gland were not, or not significantly, affected by the TK/GCV ablation approach. The DT/iDTR system allows to kill cells through apoptosis independent of cell division. In the adult mouse, we obtained a large depletion of pituitary SOX2^+^ cells, corresponding to a strong decrease in stem cell functionality as probed by pituisphere formation. The 80% ablation grade as achieved is comparable to transgenic DT-mediated stem cell ablation efficiency realised in other tissues^18–20^. Surprisingly, and counterintuitively for a stem cell population, the remaining SOX2^+^ cells did not restore the stem cell compartment to any extent. Moreover, the large SOX2^+^ cell depletion did not have any impact on the proportions of the hormonal cell populations in the adult gland. So far, no support was found that other mechanisms such as higher proliferative activity within the hormonal cell populations and/or transdifferentiation compensate for the major absence of SOX2^+^ cells in the adult gland. Alternatively, other stem cell (sub-)populations in the pituitary may take over. Cells expressing the other well-known pituitary stem cell markers SOX9 and E-cadherin were also reduced and did not repopulate. Since these markers (as well as *Cxadr*, also diminished but less pronounced) highly co-localize with SOX2 in the pituitary (^2,10,12,13^), our data do not exclude the existence of (an) additional and distinct, yet unidentified stem cell population(s) which naturally contribute(s) to endocrine cell homeostasis, or which take(s) over in the absence of the innately homeostatic-driving SOX2^+^ cells. Contribution of different stem cell populations, at least to repair after (stem cell) injury, has been described in other tissues^19,21–24^. Taken together, the bulk of SOX2^+^ cells do not need to be present to keep hormonal cell populations at normal proportions, suggesting that either the minor remaining fraction of SOX2^+^ cells is sufficient, or that SOX2^+^ cells do not play a major role in endocrine cell homeostasis in the adult gland. In the mammary gland, LGR6^+^ stem cells were also found to contribute only minimally to adult tissue homeostasis^18^.

In conditions of more active pituitary remodelling than during steady-state homeostasis, the SOX2^+^ cells remaining after DT-mediated ablation were observed to be activated. First, in the neonatal gland of DT/TAM-treated pups, proliferating SOX2^+^ cells were more abundant, and GH- or PRL-coexpressing SOX2^+^ cells increased in number. Both observations suggest that the young pituitary stem cells react to the ‘assault’ in order to rescue the early-postnatal maturation and endocrine cell development. In a second pituitary remodelling paradigm, the SOX2^+^ cells remaining after DT-mediated ablation in adult mice reacted to ADX by proliferative expansion and increased coexpression of ACTH, thereby supporting their contribution in the ACTH^+^ cell rise after ADX. Thus, the remaining 20% of the SOX2^+^ cells appear sufficient to sustain the hormonal cell response to the endocrine challenge. If we assume that the remaining fraction is also sufficient for adult homeostatic turnover (this fraction at least retains multipotent differentiation capacity *in vitro*), the following inevitable questions arise: why is the SOX2^+^ cell population so largely redundant and/or what role do the bulk of the SOX2^+^ cells play? One hypothetical explanation is that pituitary function, being critical for the body’s postnatal development and physiology, requires the presence of a sufficient ‘reserve’ of stem cells. On the other hand, the chief role of the pituitary SOX2^+^ stem cells may be situated in other domains such as repair after injury as supported by previous work^6–8^ and also found in other tissues (e.g. skin^19^ and liver^25^). Further studies are needed to clearly identify their role in regeneration after injury, requiring for instance a combined ‘SOX2^+^ lineage tracing/SOX2^+^ plus hormone^+^ cell ablation’ model. Alternatively, the cSOX2^+^ cells which do not express CreERT and thus are not hit by DT, may represent the homeostasis-driving population, however implicating that they not only generate new hormonal cells but also stem cells (with SOX2 in the nucleus) in order to maintain the stem cell population for homeostasis.

In conclusion, pituitary SOX2^+^ stem cells do not repopulate after major depletion. Repopulation is not needed for hormonal cell homeostasis and remodelling upon endocrine challenge. Answering the question on the key role of SOX2^+^ cells in the adult pituitary requires further studies, including sophisticated transgenic models.

## Methods

### Transgenic mice

Sox2^CreERT2/+^ mice (obtained from Dr. Martinez-Barbera, UCL, London) contain a CreERT2 expression cassette into the *Sox2* locus (knock-in in 1 allele), thus expressing CreERT2 under control of the endogenous *Sox2* promoter. The CreERT2 is translocated to the nucleus for activity by TAM^9^. R26^iDTR/iDTR^ mice harbour the simian DTR gene in the ROSA26 locus preceded by a LoxP-flanked, CRE-excisable STOP sequence^6–8^. Both mouse models were crossed to generate Sox2^CreERT2/+^;R26^iDTR/+^ mice. Genotyping was performed as described elsewhere^6,9^. All animals were kept in the Animal Housing Facility of the KU Leuven experiencing constant humidity, temperature and day/night cycle, and having access to water and food *ad libitum*. Animal experiments were approved by the KU Leuven Ethical Committee. All methods were performed in accordance with the relevant guidelines and regulations of the KU Leuven institutional committees.

### Treatment of mice for SOX2^+^ cell ablation

To achieve (maximal) SOX2^+^ cell ablation in the pituitary gland without evident toxicity, several time and dose injection schedules of TAM (Sigma-Aldrich, Bornem, Belgium; dissolved in corn oil) and DT (Sigma-Aldrich; dissolved in PBS) were tested in Sox2^CreERT2/+^;R26^iDTR/+^ mice (referred to as ‘Sox2/iDTR’) and their respective control Sox2^+/+^;R26^iDTR/+^ mice (referred to as ‘-/iDTR’), including: 1 TAM injection followed by a 1-day pause before 3 days of DT injection, twice daily; 2 consecutive days of TAM injection immediately followed by 3 days of DT treatment; 3 consecutive days of TAM injection followed by 2-day pause before 3 or 10 days of DT treatment; and different doses of TAM (0.15 mg and 0.30 mg TAM/g body weight). Treatment longer than 3 days with TAM resulted in morbidity most likely due to the large amounts of oil injected. Eventually, we identified a schedule of TAM injection for 3 consecutive days (0.15 mg TAM/g body weight/day), followed by a 2-day break and then 3 successive days of DT injection (4ng DT/g bodyweight, twice a day with 8-hr interval) (see Fig. 1a). No obvious external toxicity was observed in the mice, i.e. no hair or prominent weight loss, and no changes in behaviour, posture or gait. Analysis of pituitary glands was mainly performed the day immediately after the last DT injection (i.e. d9; see Fig. 1a), or 2, 4 and 6 months later (see Fig. 2a), and occasionally at intermediate time points as described. Mice of different ages were treated with TAM/DT, i.e. starting at early-postnatal age (1 or 4 weeks of age) or at adult age (8–12 weeks). In each biological replicate (indicated by ‘n’), on average 2–4 mice were used per condition. For experiments in which male and female mice were pooled, care was taken to have comparable representation of both genders.

### Pituisphere assay

The pituitary was isolated from euthanized mice, the AP separated and dispersed into single cells using trypsin as previously described^26^. To generate pituispheres, dissociated AP cells were seeded at a density of 100,000 cells/mL in 35-mm non-treated culture dishes (Corning, NY) in serum-free defined medium (SFDM; Invitrogen), which was supplemented with B27 (1/50; Life Technologies) and recombinant basic fibroblast growth factor (bFGF, 20 ng/mL; R&D Systems, Minneapolis, NE). Pituispheres were counted after 6 days in culture (3 dishes per biological replicate, meaning in total 30–90 spheres for Sox2/iDTR AP and 150–350 spheres for -/iDTR AP) either immediately fixed with paraformaldehyde (PFA, 4% in PBS; Riedel-de Haën, Seelze, Germany) for immunofluorescence (see below), or further analysed for multipotent differentiation capacity as described before^3,6,8^. In short, the clearly rounded spheres were picked manually, transferred to coverslips coated with growth factor-reduced Matrigel (1:10; BD Biosciences), and incubated in SFDM without supplements. After 7 days of culturing, spheres were fixed with PFA (4%).

### Immunofluorescence staining

Whole pituitary glands were isolated, fixed with PFA (4%) and embedded in agarose or paraffin as previously described^27^. For immunofluorescence analysis of agarose-embedded pituitaries, coronal vibratome sections (45 μm) were permeabilised with 0.4% Triton X-100 (Sigma-Aldrich) and incubated overnight with (a combination of) primary antibodies (see Supplementary Table S2). Next, sections were incubated with (a combination of) secondary antibodies (see Supplementary Table S2), and nuclei were labelled with ToPro3 (Life Technologies), all as described before^6–8,16,26,27^. Immunofluorescence was examined using a Zeiss LSM 510 confocal laser-scanning microscope (Zeiss, Zaventem, Belgium) accessible through the Cell Imaging Core (CIC; KU Leuven). Z-stacks were collected and analysed using Zeiss LSM Image Browser and ImageJ (http://imagej.nih.gov/ij/), and pictures were prepared using the same programs and Microsoft PowerPoint (2016). H&E staining was performed on paraffin-embedded pituitary sections (5 μm) as described^28^. The forestomach was isolated, fixed in 10% neutral-buffered formalin and embedded in paraffin. Immunofluorescence staining of paraffin-embedded pituitary and forestomach sections was performed after antigen retrieval with citrate buffer (pH 6) and permeabilisation with Triton X-100 (0.1% in PBS). Following incubation with primary and secondary antibodies (see Supplementary Table S2), sections were covered with Vectashield (containing 4′,6-diamidino-2-phenylindole or DAPI; Vector Laboratories) and analysed with a Leica DM5500 epifluorescence microscope (Leica Microsystems, Diegem, Belgium) accessible through InfraMouse (KU Leuven-VIB). Recorded images were converted to pictures with ImageJ and Microsoft PowerPoint (2016).

Cytospin samples of 50,000 dissociated AP cells each and pituispheres were fixed with PFA (4%), permeabilised with saponin (0.25%; Sigma-Aldrich), and further immunostained for SOX2, the proliferation marker Ki67 and/or hormones (see Supplementary Table S2) as described^6–8,16,27^. Nuclei were visualized using Vectashield containing DAPI. To enumerate the respective immunopositive cells, pictures were captured using the Leica DM5500 microscope, and cells counted with ImageJ. For the counting of the SOX2^+^ cells, 3 cytospin slides were immunostained per biological replicate, and cells were counted in 10 images per cytospin slide using 40X magnification (in total, 1500–3000 cells were counted per biological replicate). For the counting of the hormone^+^ cells, immunostaining was performed on 2 cytospin slides, and cells were counted in 3–6 images per cytospin slide using 20X magnification (in total, 2500–5000 cells were counted per biological replicate). The SOX2^+^ cell ablation grade was calculated using the following formula: % SOX2^+^ cell ablation = [(average % SOX2^+^ cells in -/iDTR AP – average % SOX2^+^ cells in Sox2/iDTR AP)/average % SOX2^+^ cells in -/iDTR AP]*100. Unless otherwise stated, data from male and female mice were pooled since no differences were observed in SOX2^+^ cell ablation.

### Adrenalectomy

Adult mice were injected with TAM/DT (see Fig. 1a), and bilateral ADX (-/iDTR: n = 5, Sox2/iDTR: n = 4), or SHAM operation (-/iDTR: n = 3, Sox2/iDTR: n = 3) was performed under ketamine/xylazine anaesthesia 4 days later. Mice were given sodium chloride (0.9%) in the drinking water after surgery, and pituitaries were harvested for analysis 7 days after surgery.

### Gene expression analysis by RT-qPCR

Total RNA was extracted from pituitaries with the RNeasy Micro kit (Qiagen), reverse-transcribed using the Superscript III First-Strand Synthesis Supermix (Invitrogen) and subjected to SYBR Green-based quantitative real-time PCR (qPCR) using the StepOnePlus Real-Time PCR System (AB Applied Biosystems). Forward and reverse primers (see Supplementary Table S3) were designed using PerlPrimer^29^ and PrimerBLAST (https://www.ncbi.nlm.nih.gov/tools/primer-blast/). Glyceraldehyde-3-phosphate dehydrogenase (*Gapdh*) and β-actin (*ActB*) were used as housekeeping genes. Relative gene expression levels were calculated as ∆Ct values (Ct ‘target’ minus Ct ‘average housekeeping genes’) and compared between Sox2/iDTR and -/iDTR to express fold change, i.e. 2^−(ΔCt (Sox2/iDTR) − ΔCt (−/iDTR))^.

### Statistical analysis

Normal distribution was verified with Shapiro-Wilk normality test using GraphPad Prism (Version 7.03) before performing statistical analyses. In case of normal distribution, unpaired two-tailed t-student test was applied for comparison of 2 groups. When normal distribution was not achieved, Mann–Whitney U test was applied for comparison of 2 groups. In case of multiple comparisons (more than 2 groups), 1-way analysis of variance (ANOVA) was performed followed by Tuckey test for Multiple Comparison (data were normally distributed). Statistical analyses were performed (in case of n ≥ 3) using GraphPad Prism (Version 7.03) and Microsoft Excel software (2016). Statistical significance was defined as p < 0.05. Exact p-values are provided in Supplementary Table S1.

## Electronic supplementary material

Supplementary Figures and Tables
