# Supplementary material for: Major depletion of SOX2+ stem cells in the adult pituitary is not restored which does not affect hormonal cell homeostasis and remodelling
Source: Sci Rep. 2017 Dec 5;7:16940. doi: 10.1038/s41598-017-16796-2 (PMC5717068; doi:10.1038/s41598-017-16796-2)
Supplement: Supplementary file 1 — Supplementary Figures and Tables [file 41598_2017_16796_MOESM1_ESM.pdf]

Major depletion of $SOX2^+$ stem cells in the adult pituitary is not restored which does not affect hormonal cell homeostasis and remodelling

Heleen Roose, Benoit Cox, Matteo Boretto, Conny Gysemans, Annelies Vennekens, Hugo Vankelecom

Supplementary Fig. S1

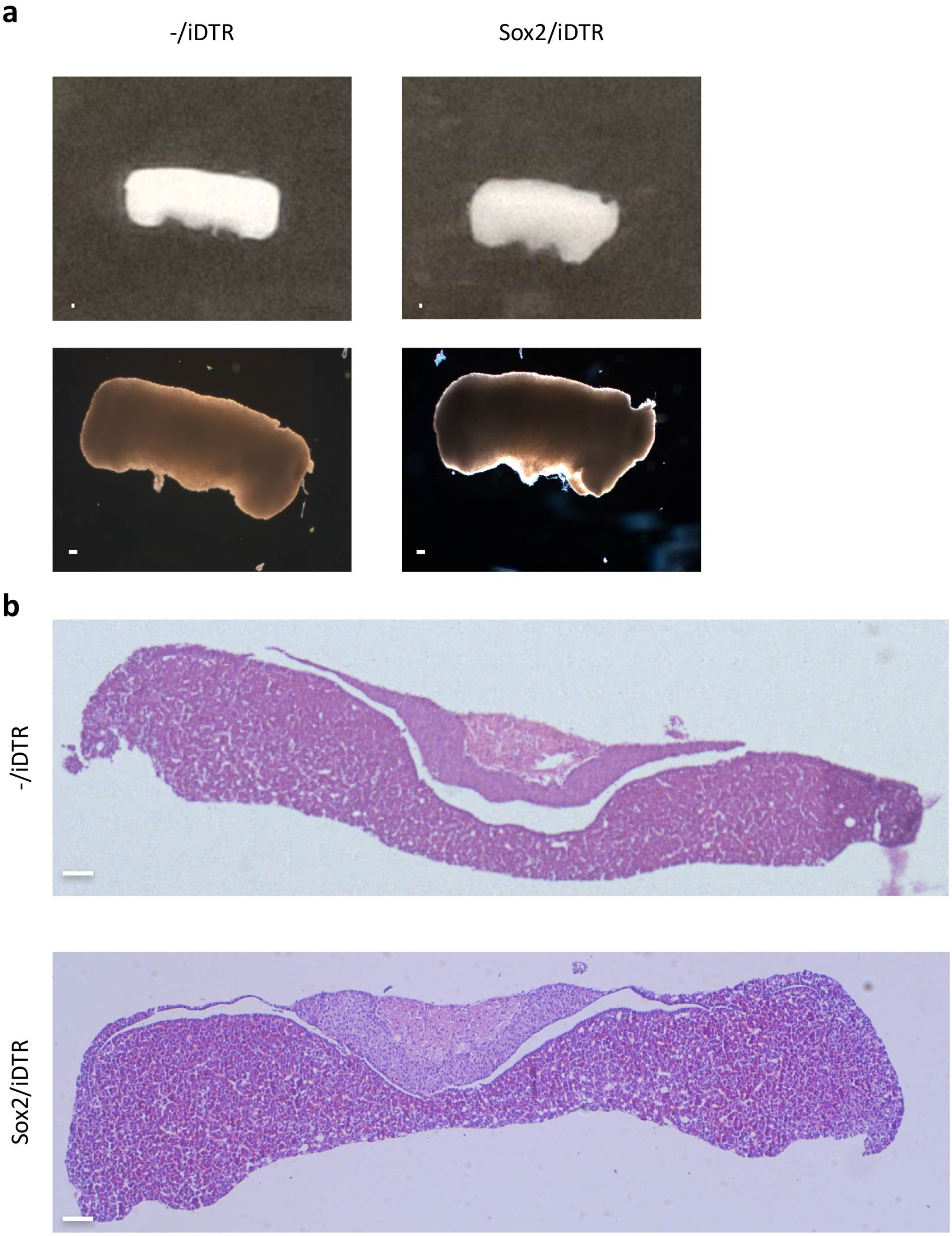

Supplementary Fig. S1. Pituitary morphology after SOX2+ cell ablation in adult mice

a. Microscopic brightfield pictures of the pituitary of adult -/iDTR control and Sox2/iDTR mice at d9 after TAM/DT treatment. Representative pictures are shown. Scale bar: 50 µm.

b. H&E-stained-paraffin sections of adult -/iDTR control and Sox2/iDTR mice at d9 after TAM/DT treatment. Representative pictures are shown. Scale bar: 50 µm.

Supplementary Fig. S2

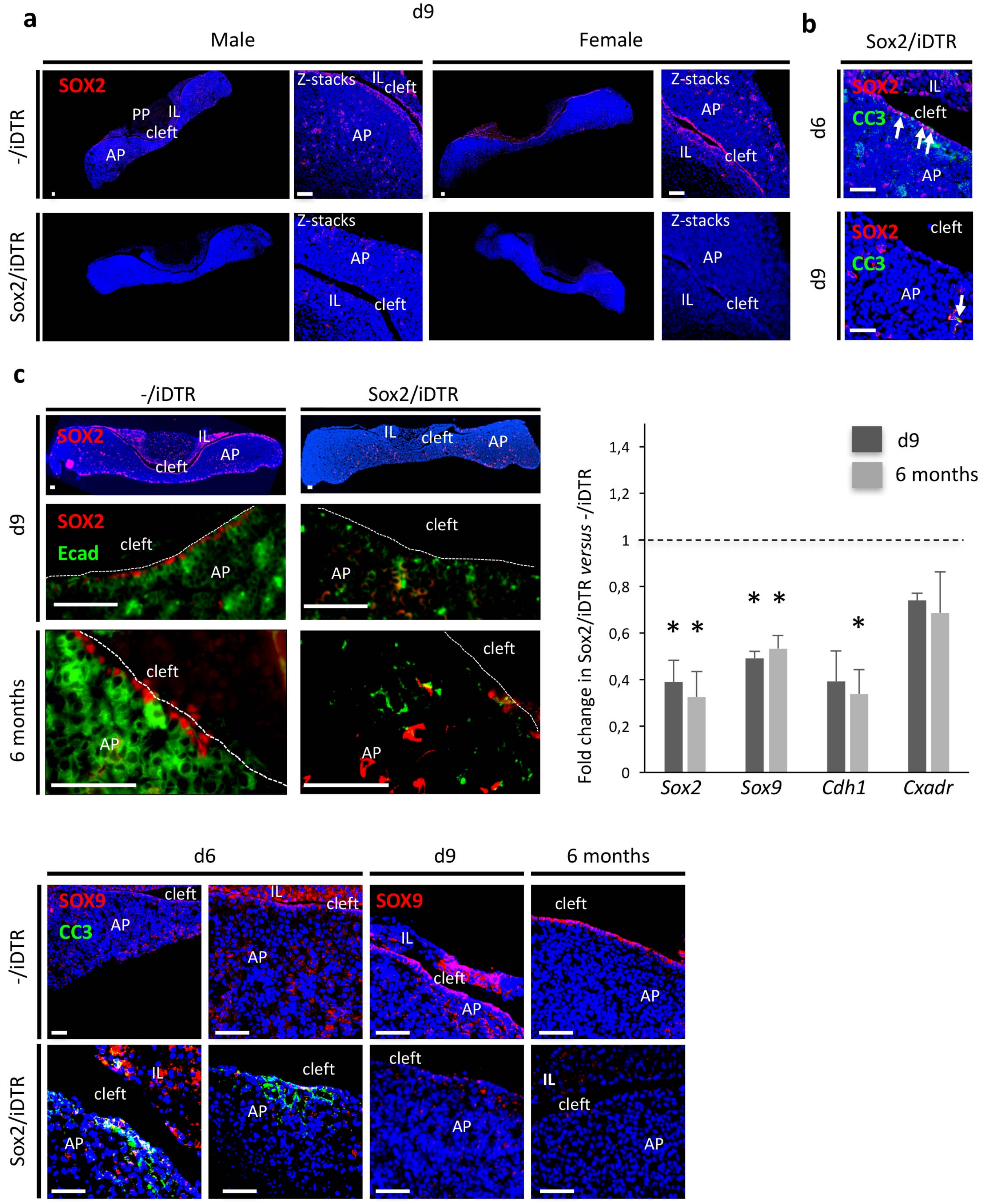

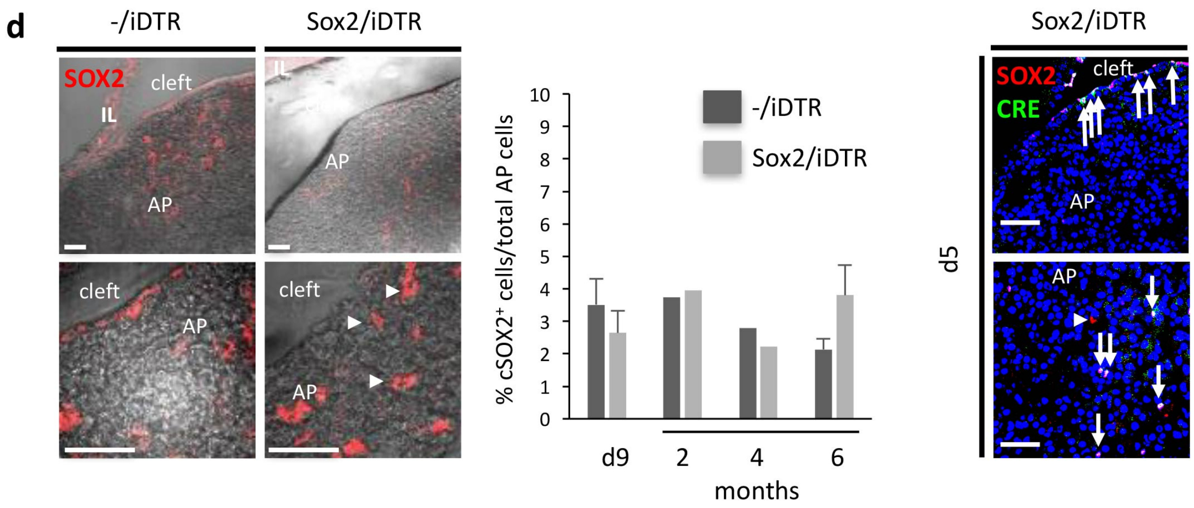

Supplementary Fig. S2. Pituitary phenotype after $SOX2^{+}$ cell ablation in adult mice

a. Overviews and higher magnifications showing a 2D projection of compiled confocal z-stacks (as indicated) of pituitary vibratome sections isolated from adult -/iDTR and Sox2/iDTR mice injected with TAM/DT and analysed for SOX2 (red) immediately after treatment (d9). Nuclei are labelled with TOPRO3 (blue). Representative pictures are shown. Scale bar: 50 µm. AP, anterior pituitary; IL, intermediate lobe; PP, posterior pituitary.

b. Pituitary vibratome sections isolated from adult Sox2/iDTR mice injected with TAM/DT and analysed for SOX2 (red) and CC3 (green) after 1 day of DT (d6; see Fig. 1a) and after full treatment (d9). Nuclei are labelled with TOPRO3 (blue). Representative pictures are shown. Arrows indicate double-immunopositive cells. Scale bar: 50 µm.

c. *Upper left*: Pituitary paraffin sections isolated from adult -/iDTR and Sox2/iDTR mice injected with TAM/DT and analysed for SOX2 (red) and E-cadherin (Ecad; green) immediately after treatment (d9) and 6 months later. Nuclei are labelled with DAPI (blue). Representative pictures are shown. Scale bar: 50 µm.

Upper right: Gene expression analysis of the pituitary stem cell markers as indicated in the AP of adult Sox2/iDTR mice injected with TAM/DT and analysed immediately after treatment (d9) and 6 months later, shown as fold difference *versus* expression in the AP of -/iDTR mice (set as 1; dotted line). Bar represents mean ± SEM (n=3). $*p< 0.05$.

Lower: Pituitary vibratome sections isolated from adult -/iDTR and Sox2/iDTR mice injected with TAM/DT and analysed for SOX9 (red) and CC3 (green) after 1 day of DT (d6; see Fig. 1a), and for SOX9 alone (red) immediately after full treatment (d9) and 6 months later. Nuclei are labelled with TOPRO3 (blue). Representative pictures are shown. Scale bar: 50 µm.

d. *Left*: Combined immunofluorescence/brightfield-contrast pictures of pituitary vibratome sections isolated from adult -/iDTR and Sox2/iDTR mice injected with TAM/DT and analysed for SOX2 (red) immediately after treatment (d9). Representative pictures are shown. Scale bar: 50 µm. Surviving $SOX2^{+}$ cells with immunoreactive signal in the cytoplasm ($cSOX2^{+}$ cells) are indicated (arrowheads).

Middle: Proportion of $cSOX2^{+}$ cells as quantified in immunostained AP cells dispersed from adult -/iDTR control (dark grey) and Sox2/iDTR mice (light grey) injected with TAM/DT (see Fig. 1a) and analysed 1 day (d9) and 2, 4 and 6 months later. Bars represent mean (± SEM for n≥3) (d9: n=4; 2 months: n=1; 4 months: n=2; 6 months: n=4). Differences (as analysed when n≥3, i.e. for d9 and 6 months) are non-significant (see Supplementary Table S1).

Right: Pituitary vibratome sections isolated from adult Sox2/iDTR mice injected for 3 days with TAM and analysed for SOX2 (red) and CRE (green) (d5, see Fig. 1a). Nuclei are labelled with TOPRO3 (blue). Representative pictures are shown. Scale bar: 50 µm. Double $nSOX2^{+}/CRE^{+}$ are indicated (arrows). $cSOX2^{+}$ cells do not express CRE (arrowhead).

Supplementary Fig. S3

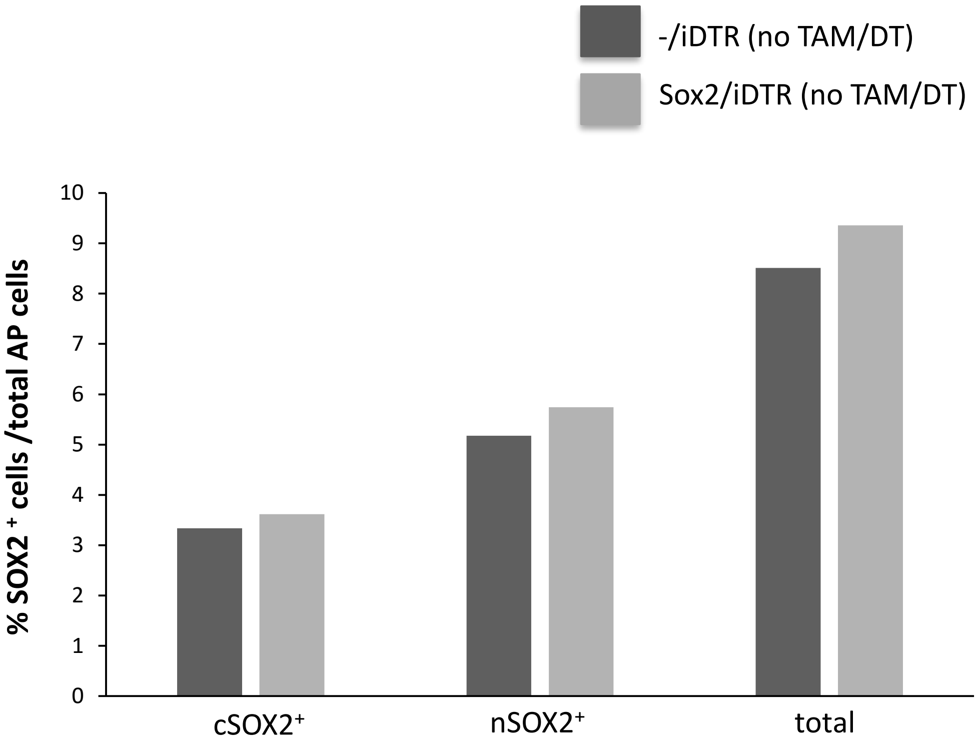

Supplementary Fig. S3. *Sox2* hemizygosity has no impact on pituitary Sox2⁺ cell number
Proportion of SOX2⁺ cells in the AP as quantified in immunostained AP cell cytospin samples from adult -/iDTR control mice (dark grey) and Sox2/iDTR mice (light grey) (no TAM/DT treatment). Bars represent mean (n=2).

Supplementary Fig. S4

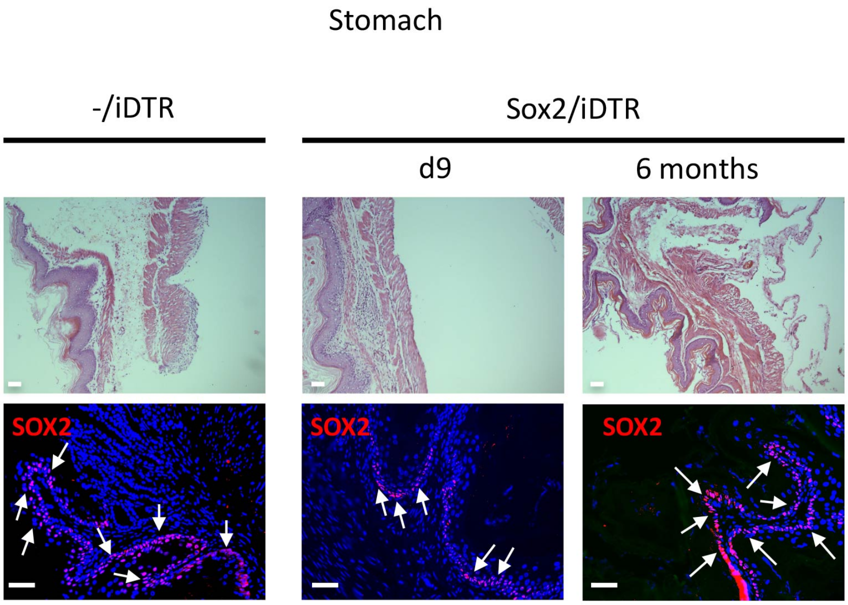

Supplementary Fig. S4. SOX2$^+$ cell ablation and regeneration in the stomach of adult mice
H&E staining (upper) and immunofluorescent analysis (lower) for SOX2 (red) in paraffin sections of the stomach from adult -/iDTR control and Sox2/iDTR mice injected with TAM/DT and analysed for SOX2 (red) immediately after treatment (d9) and 6 months later. SOX2$^+$ cells (arrows) are considerably ablated but repopulate. Representative pictures of the forestomach are shown, the nucleus being labelled with TOPRO3 (blue). Scale bar: 50 µm.

Supplementary Fig. S5

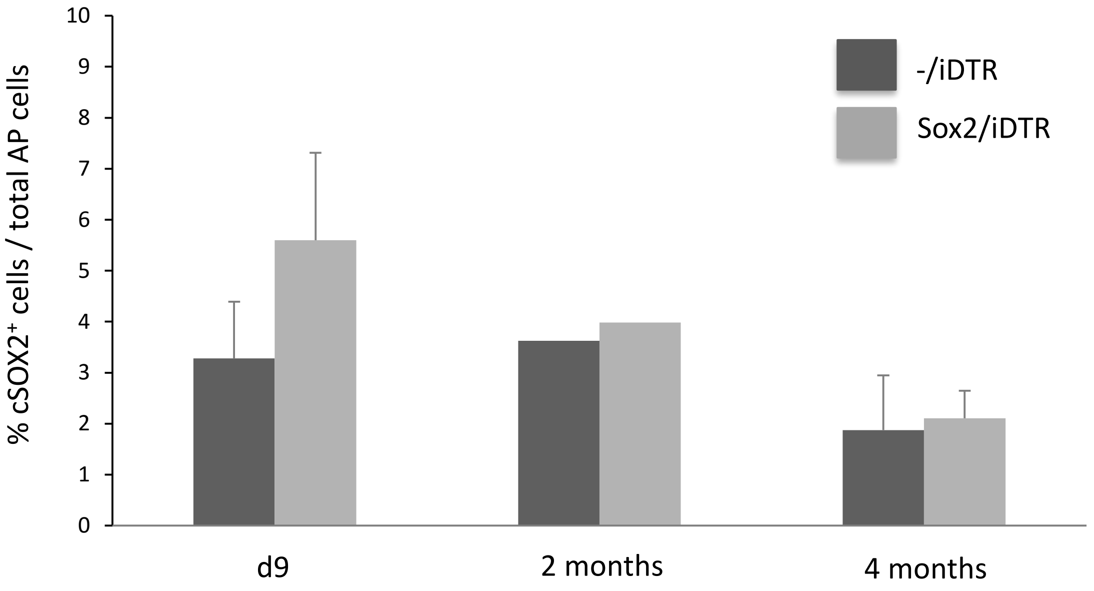

Supplementary Fig. S5. Cytoplasmic SOX2$^+$ cells after SOX2$^+$ cell ablation in 4-week-old mice
Proportion of cSOX2$^+$ cells as quantified in immunostained AP cells dispersed from 4-week-old -/iDTR control (dark grey) and Sox2/iDTR mice (light grey) injected with TAM/DT and analysed 1 day (d9) and 2 and 4 months later. Bars represent mean (± SEM for $n \geq 3$) (d9: n=4; 2 months: n=1; 4 months: n=3). Differences (as analysed when $n \geq 3$, i.e. for d9 and 4 months) are non-significant (see Supplementary Table S1).

Supplementary Fig. S6

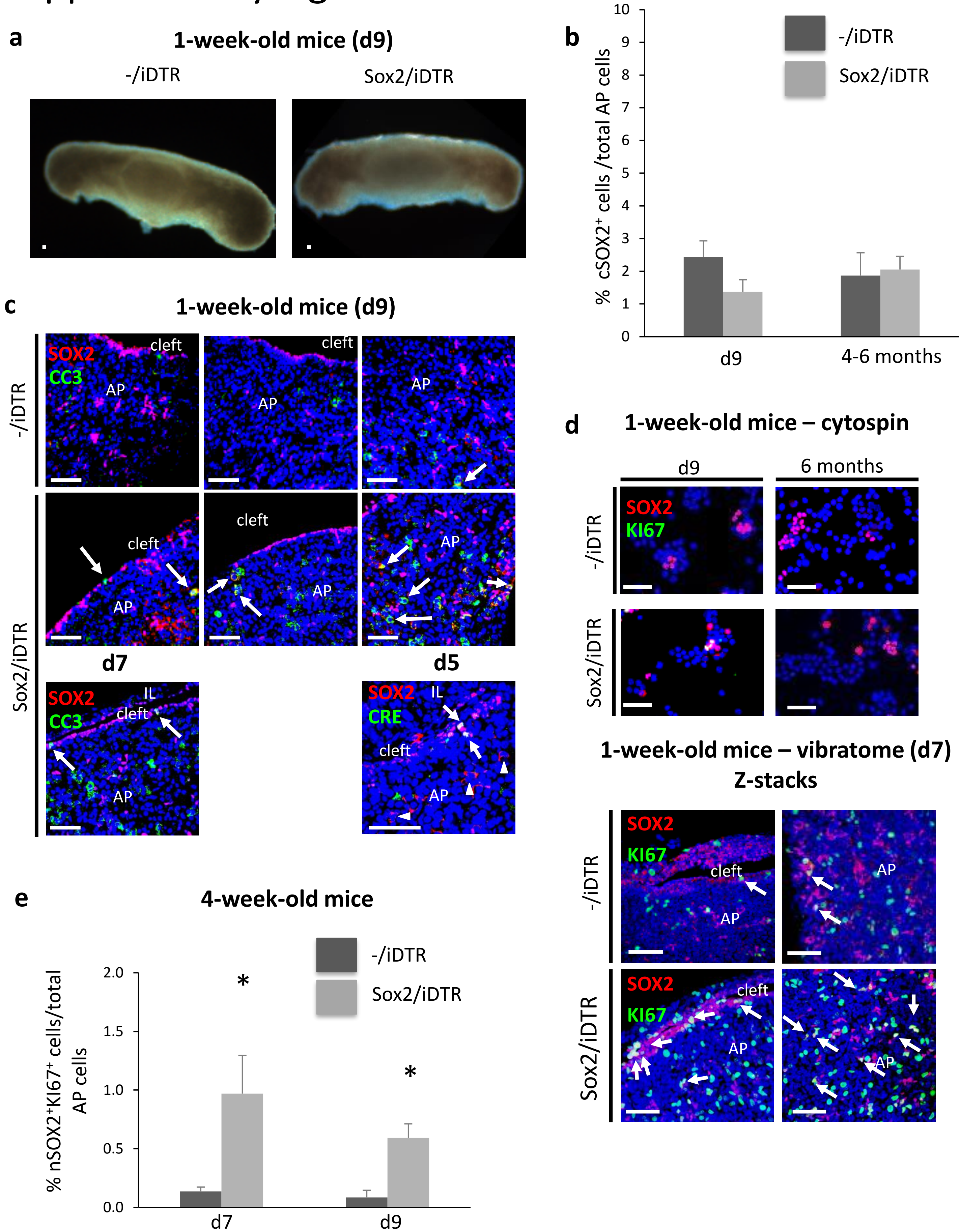

Supplementary Fig. S6. Pituitary phenotype after SOX2$^+$ cell ablation in early-postnatal mice
a. Microscopic brightfield pictures of the pituitary of neonatal -/iDTR control and Sox2/iDTR mice at d9 after TAM/DT treatment. Representative pictures are shown. Scale bar: 50 µm.
b. Proportion of cSOX2$^+$ cells as quantified in immunostained AP cells dispersed from neonatal -/iDTR control (dark grey) and Sox2/iDTR mice (light grey) injected with TAM/DT and analysed 1 day (d9) and 4-6 months later. Bars represent mean ± SEM (n=3). Differences are non-significant (see Supplementary Table S1).
c. Pituitary vibratome sections isolated from -/iDTR and Sox2/iDTR mice injected at 1 week of age with TAM/DT and analysed for SOX2 (red) and the apoptotic marker CC3 (green) after full treatment (d9) and at d7 (i.e. after the first day of DT injection, see Fig. 1a), and analysed for SOX2 (red) and CRE (green) after TAM treatment (d5). Representative pictures are shown, the nucleus being labelled with TOPRO3 (blue). Scale bar: 50 µm. Double nSOX2$^+$/CC3$^+$ cells or nSOX2$^+$/CRE$^+$ are indicated (arrows). cSOX2$^+$ cells do not express CRE (arrowheads).
d. *Upper*: Cytospin samples of AP cells immunostained for SOX2 (red) and Ki67 (green) from 1-week-old -/iDTR control mice and Sox2/iDTR mice immediately after DT treatment (d9) and 6 months later. Nuclei are labelled with DAPI (blue). Representative pictures are shown. Scale bar: 50 µm.
Lower: Pituitary vibratome sections isolated from -/iDTR and Sox2/iDTR mice injected at 1 week of age with TAM/DT and analysed for SOX2 (red) and Ki67 (green) at d7 (i.e. after the first day of DT injection, see Fig. 1a). A 2D projection of compiled confocal z-stacks corresponding to the pictures shown in Fig. 3d are presented, the nucleus being labelled with TOPRO3 (blue). Representative pictures are shown. Scale bar: 50 µm. Double nSOX2$^+$/Ki67$^+$ cells are indicated (arrows).
e. Proportion of Ki67$^+$/nSOX2$^+$ in the AP in TAM/DT-injected 4-week-old Sox2/iDTR mice (light grey) as compared to -/iDTR control mice (dark grey), analysed 1 day after start of DT injection (d7) and after full-term DT injection (d9). Bars represent mean ± SEM (n=3).

Supplementary Fig. S7

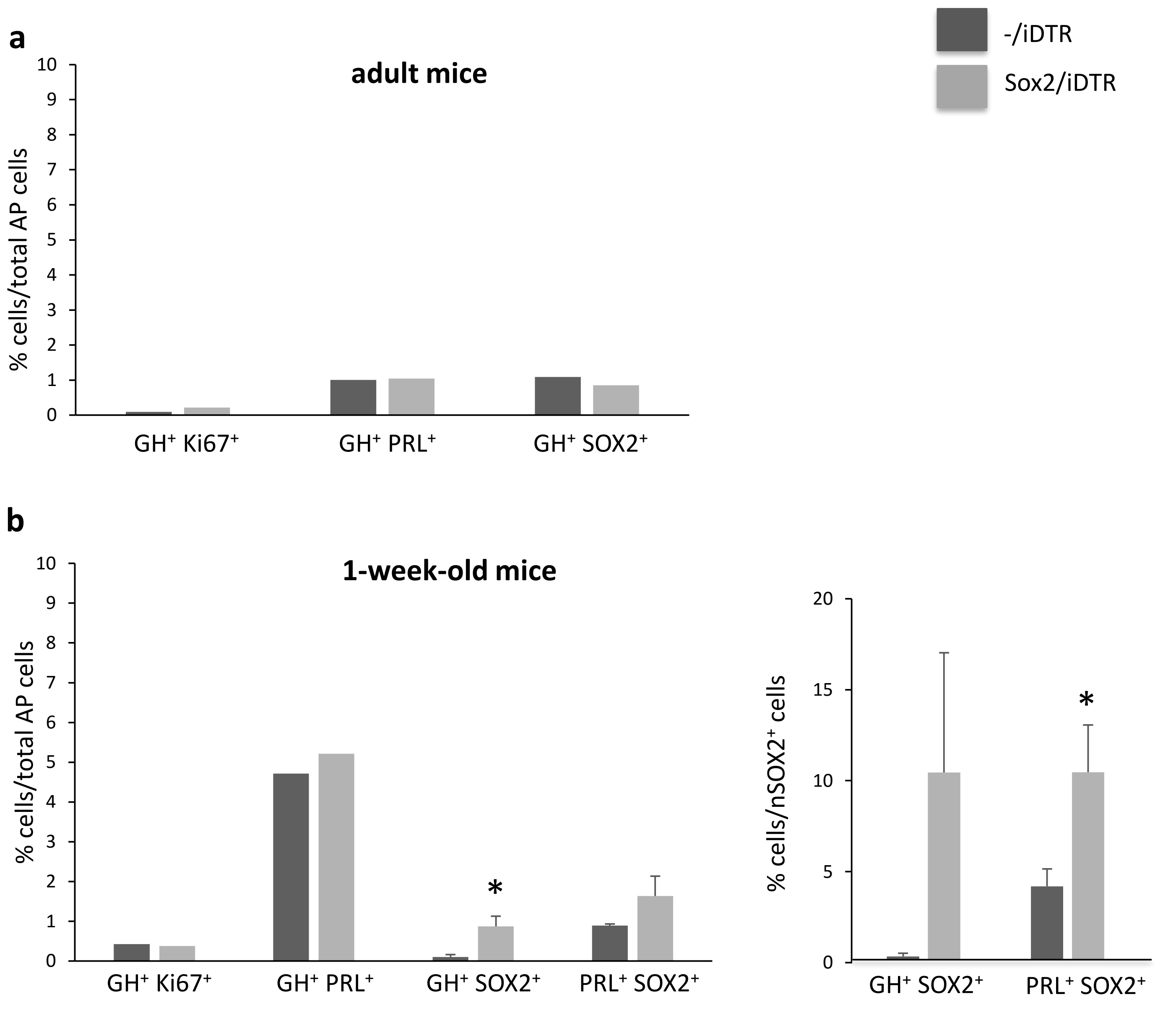

Supplementary Fig. S7. Proliferation and transdifferentiation of hormonal cells, and SOX2/hormone co-expression after SOX2$^+$ cell ablation in adult and neonatal mice

a. Proportion of double-immunopositive cells as indicated in the AP of TAM/DT-injected adult Sox2/iDTR mice (light grey) as compared to -/iDTR control mice (dark grey), analysed 1 week after last DT injection (d16). Bars represent represent mean (n=2).

b. *Left:* Proportion of double-immunopositive cells as indicated in the AP of TAM/DT-injected 1-week-old Sox2/iDTR mice (light grey) as compared to -/iDTR control mice (dark grey), analysed 1 day after last DT injection (d9). Bars represent mean (± SEM for n≥3) (PRL$^+$SOX2$^+$, GH$^+$SOX2$^+$: n=3; GH$^+$Ki67$^+$, GH$^+$PRL$^+$: n=1). *, $p<0.05$.

Right: Bar graph showing the proportion of PRL$^+$SOX2$^+$ and GH$^+$SOX2$^+$ within the nSOX2$^+$ cell population (mean ± SEM; n=3) in the AP of TAM/DT-injected 1-week-old Sox2/iDTR mice (light grey) as compared to -/iDTR control mice (dark grey), analysed immediately after TAM/DT injection (d9). *, $p<0.05$.

Supplementary Table S1: Exact p-values from statistical analyses

Figure	Comparison	P value
Fig. 1c	Ablation in adult: analysis d9 $nSOX2^+$ SOX2/iDTR vs -/iDTR	0.004
	Ablation in adult: analysis d9 $iSphere^+$ SOX2/iDTR vs -/iDTR	0.031
Fig. 2b	Ablation in adult: analysis d9 vs 6 months in Sox2/iDTR	0.689
Fig. 2d	Ablation in adult: analysis 6 months $iSphere^+$ SOX2/iDTR vs -/iDTR	0.014
Fig. 3a	Ablation in 4-week-old: analysis d9 $nSOX2^+$ SOX2/iDTR vs -/iDTR	0.005
	Ablation in 4-week-old: analysis 4 months $nSOX2^+$ SOX2/iDTR vs -/iDTR	0.008
	Ablation in 4-week-old: $nSOX2^+$ in SOX2/iDTR d9 vs 4 months	0.196
Fig. 3b	% $nSOX2^+$ cell ablation analysis d9: age of start ablation 1 week vs 4 weeks	0.005
	% $nSOX2^+$ cell ablation analysis d9: age of start ablation 1 week vs adult	0.000
	% $nSOX2^+$ cell ablation analysis d9: age of start ablation 4 weeks vs adult	0.005
Fig. 3c	Ablation in 1-week-old: analysis d9 $nSOX2^+$ SOX2/iDTR vs -/iDTR	0.002
	Ablation in 1-week-old: analysis 4-6 months $nSOX2^+$ SOX2/iDTR vs -/iDTR	0.049
	Ablation in 1-week-old: $nSOX2^+$ in SOX2/iDTR d9 vs 4-6 months	0.679
Fig. 3d	Ablation in 1-week-old: analysis d9 % $Ki67^+nSOX2^+$/total $nSOX2^+$ cells SOX2/iDTR vs -/iDTR	0.034
Fig. 4a	Ablation in adult: analysis d9 αGSU^+ SOX2/iDTR vs -/iDTR (Female)	0.330
	Ablation in adult: analysis d9 $ACTH^+$ SOX2/iDTR vs -/iDTR (Female)	0.057
	Ablation in adult: analysis d9 GH^+ SOX2/iDTR vs -/iDTR (Female)	0.543
	Ablation in adult: analysis d9 PRL^+ SOX2/iDTR vs -/iDTR (Female)	0.324
	Ablation in adult: analysis d9 αGSU^+ SOX2/iDTR vs -/iDTR (Male)	0.827
	Ablation in adult: analysis d9 $ACTH^+$ SOX2/iDTR vs -/iDTR (Male)	0.509
	Ablation in adult: analysis d9 GH^+ SOX2/iDTR vs -/iDTR (Male)	0.901
	Ablation in adult: analysis d9 PRL^+ SOX2/iDTR vs -/iDTR (Male)	0.973
	Ablation in adult: analysis 6 months αGSU^+ SOX2/iDTR vs -/iDTR (Female)	0.489
	Ablation in adult: analysis 6 months $ACTH^+$ SOX2/iDTR vs -/iDTR (Female)	0.121
	Ablation in adult: analysis 6 months GH^+ SOX2/iDTR vs -/iDTR (Female)	0.956
	Ablation in adult: analysis 6 months PRL^+ SOX2/iDTR vs -/iDTR (Female)	0.225
	Ablation in adult: analysis 6 months αGSU^+ SOX2/iDTR vs -/iDTR (Male)	0.809
	Ablation in adult: analysis 6 months $ACTH^+$ SOX2/iDTR vs -/iDTR (Male)	0.575
	Ablation in adult: analysis 6 months GH^+ SOX2/iDTR vs -/iDTR (Male)	0.924
	Ablation in adult: analysis 6 months PRL^+ SOX2/iDTR vs -/iDTR (Male)	0.428
Fig. 4b	Ablation in 1-week-old: analysis d9 αGSU^+ SOX2/iDTR vs -/iDTR (Female)	0.734
	Ablation in 1-week-old: analysis d9 $ACTH^+$ SOX2/iDTR vs -/iDTR (Female)	0.115

	Ablation in 1-week-old: analysis d9 GH$^+$ SOX2/iDTR vs -/iDTR (Female)	0.253
	Ablation in 1-week-old: analysis d9 PRL$^+$ SOX2/iDTR vs -/iDTR (Female)	0.503
	Ablation in 1-week-old: analysis d9 αGSU$^+$ SOX2/iDTR vs -/iDTR (Male)	0.305
	Ablation in 1-week-old: analysis d9 ACTH$^+$ SOX2/iDTR vs -/iDTR (Male)	0.212
	Ablation in 1-week-old: analysis d9 GH$^+$ SOX2/iDTR vs -/iDTR (Male)	0.806
	Ablation in 1-week-old: analysis d9 PRL$^+$ SOX2/iDTR vs -/iDTR (Male)	0.867
Fig. 4c	Ablation in 1-week-old: analysis 6 months αGSU$^+$ SOX2/iDTR vs -/iDTR (Pooled)	0.494
	Ablation in 1-week-old: analysis 6 months ACTH$^+$ SOX2/iDTR vs -/iDTR (Pooled)	0.829
	Ablation in 1-week-old: analysis 6 months GH$^+$ SOX2/iDTR vs -/iDTR (Pooled)	0.991
	Ablation in 1-week-old: analysis 6 months PRL$^+$ SOX2/iDTR vs -/iDTR (Pooled)	0.647
Fig. 4d	ADX: ACTH$^+$ ADX vs SHAM	0.032
	ADX: nSOX2$^+$ ADX vs SHAM	0.072
	ADX: KI67$^+$ nSOX2$^+$ ADX vs SHAM	0.498
	ADX: ACTH$^+$ nSOX2$^+$ ADX vs SHAM	0.970
	ADX: ACTH$^+$ ADX Sox2/iDTR vs SHAM	0.001
	ADX: nSOX2$^+$ ADX Sox2/iDTR vs SHAM	0.017
	ADX: KI67$^+$ nSOX2$^+$ ADX Sox2/iDTR vs SHAM	0.480
	ADX: ACTH$^+$ nSOX2$^+$ ADX Sox2/iDTR vs SHAM	0.026
	ADX: ACTH$^+$ SHAM Sox2/iDTR vs SHAM	0.970
	ADX: nSOX2$^+$ SHAM Sox2/iDTR vs SHAM	0.017
	ADX: nSOX2$^+$ ADX Sox2/iDTR vs SHAM Sox2/iDTR	0.025
Supplementary Fig. S2c	Ablation in adult: analysis d9: gene expression *Sox2* SOX2/iDTR vs -/iDTR	0.037
	Ablation in adult: analysis d9: gene expression *Sox9* SOX2/iDTR vs -/iDTR	0.023
	Ablation in adult: analysis d9: gene expression *Cdh1* SOX2/iDTR vs -/iDTR	0.100
	Ablation in adult: analysis d9: gene expression *Cxadr* SOX2/iDTR vs -/iDTR	0.224
	Ablation in adult: analysis 6 months: gene expression *Sox2* SOX2/iDTR vs -/iDTR	0.015
	Ablation in adult: analysis 6 months: gene expression *Sox9* SOX2/iDTR vs -/iDTR	0.026
	Ablation in adult: analysis 6 months: gene expression *Cdh1* SOX2/iDTR vs -/iDTR	0.042
	Ablation in adult: analysis 6 months: gene expression *Cxadr* SOX2/iDTR vs -/iDTR	0.229
Supplementary Fig. S2d	Ablation in adult: analysis d9 cSOX2$^+$ SOX2/iDTR vs -/iDTR	0.686
	Ablation in adult: analysis 6 months cSOX2$^+$ SOX2/iDTR vs -/iDTR	0.140
Supplementary Fig. S5	Ablation in 4-week-old: analysis d9 cSOX2$^+$ SOX2/iDTR vs -/iDTR	0.300
	Ablation in 4-week-old: analysis 4 months cSOX2$^+$ SOX2/iDTR vs -/iDTR	0.860
Supplementary Fig. S6b	Ablation in 1-week-old: analysis d9 cSOX2$^+$ SOX2/iDTR vs -/iDTR	0.818

	Ablation in 1-week-old: analysis 4-6 months cSOX2$^+$ SOX2/iDTR vs -/iDTR	0.862
Supplementary Fig. S6e	Ablation in 4-week-old: analysis d7 % Ki67$^+$nSOX2$^+$ SOX2/iDTR vs -/iDTR	0.032
	Ablation in 4-week-old: analysis d9 % Ki67$^+$nSOX2$^+$ SOX2/iDTR vs -/iDTR	0.012
Supplementary Fig. S7	Ablation in 1-week-old: analysis d9 % GH$^+$SOX2$^+$ SOX2/iDTR vs -/iDTR	0.020
	Ablation in 1-week-old: analysis d9 % PRL$^+$SOX2$^+$ SOX2/iDTR vs -/iDTR	0.096
	Ablation in 1-week-old: analysis d9 % GH$^+$SOX2$^+$/SOX2$^+$ cells SOX2/iDTR vs -/iDTR	0.099
	Ablation in 1-week-old: analysis d9 % PRL$^+$SOX2$^+$/SOX2$^+$ cells SOX2/iDTR vs -/iDTR	0.032

Supplementary Table S2: Antibodies used for immunofluorescence staining

Antibody	Concentration	Application[a]	Company/ Supplier
**Primary antibodies**			
Goat anti-human SOX2	1/250-1/750	C/V/P/S	Immune Systems, Devon, UK
Goat anti-human SOX9	1/20	V	R&D Systems, Minneapolis, NE
Rabbit anti-human Ki67	1/50-1/100	C/V	Thermo Scientific, Fremonet, CA
Rabbit anti-human CC3	1/100	V	Merck Millipore, Darmstadt, Germany
Rabbit anti-human E-Cadherin	1/400	P	Cell Signaling Technology, Danvers, MA
Guinea pig anti-rat PRL	1/2500	C/V/S	Dr. A.F Parlow, NHPP, Harbor-UCLA Medical Center, Torrance, CA
Guinea pig anti-rat GH	1/5000	C/V/S	
Guinea pig anti-rat αGSU	1/1000	C/V/S	
Rabbit anti-rat ACTH	1/5000	C/V/S	
Rabbit anti-rat GH	1/10000	C/V	
**Secondary antibodies**			
Donkey anti-goat Alexa Fluor 555	1/1000	C/V/P/S	Life Technologies, Grand Island, NY
Donkey anti-rabbit Alexa Fluor 488			
Donkey anti-rabbit Alexa Fluor 555			
Donkey anti-guinea pig FITC		C/V/S	Jackson Immuno Research, West Grove, PA
Donkey anti-guinea pig Cy3			

[a]C: cytospin samples, V: vibratome sections, P: paraffin sections, S: spheres

Supplementary Table S3: Primers for qPCR

Gene Name	Symbol	Species	Forward (5'-3')	Reverse (5'-3')
sex determining region Y)-box 2	*Sox2*	mouse	CTGTTTTTTCATCCCAATTGCA	CGGAGATCTGGCGGAGAATA
sex determining region Y)-box 9	*Sox9*	mouse	CTCCTAATGCTATCTTCAAGGCG	ACCCTGAGATTGCCCAGAG
cadherin 1	*Cdh1*	mouse	AGAAGATCACGTATCGGATTTGG	TTCTTCACATGCTCAGCGTC
coxsackie virus and adenovirus receptor	*Cxadr*	mouse	CTACTGTGCTTCGTGCTCTTG	TTTTTCGATCCTCTGTTCGGG
glyceraldehyde-3-phosphate dehydrogenase	*Gapdh*	Mouse	TCGGTGTGAACGGATTTGG	CGTGAGTGGAGTCATACTGG
β–actin	*ActB*	mouse	GCTGAGAGGGAAATCGTGCGTG	CCAGGGAGGAAGAGGATGCGG
